# Stay Away From the Cold: An Unusual Case of Cold Agglutinin Disease Presenting as Recurrent Transient Ischemic Attacks

**DOI:** 10.7759/cureus.73702

**Published:** 2024-11-14

**Authors:** Francisco Gonçalves, Daniela Duarte, Filipa Reis, Alexandra Vaz, Ana Gomes

**Affiliations:** 1 Internal Medicine, Centro Hospitalar Tondela-Viseu, Viseu, PRT; 2 Stroke Unit, Centro Hospitalar Tondela-Viseu, Viseu, PRT

**Keywords:** arterial thromboembolic events, autoimmune hemolytic anemia (aiha), cold agglutinin disease, cold agglutinins, transient ischemic attacks

## Abstract

Cold agglutinin disease (CAD) is a rare autoimmune hemolytic anemia caused by cold-reactive IgM antibodies leading to complement-mediated hemolysis. While CAD-associated venous thromboembolism is recognized, its role in arterial thromboembolic events, particularly ischemic stroke, is poorly defined.

We report an 84-year-old woman who developed acute onset upper left extremity weakness following exposure to sub-zero temperatures. She had a history of similar transient episodes during cold seasons and recurrent anemia. Physical examination revealed decreased muscle strength in the upper left extremity (Grade 4+ out of 5) and scleral icterus. Laboratory findings showed normocytic anemia, elevated reticulocytes, high lactate dehydrogenase, indirect hyperbilirubinemia, and undetectable haptoglobin levels. Peripheral smear showed erythrocyte agglutination. Direct antiglobulin test was positive for C3d and IgM, confirming CAD. Vascular imaging showed significant atherosclerotic disease with near-occlusive (>80%) stenosis of the right internal carotid artery.

The patient's transient ischemic attacks were attributed to compromised cerebral perfusion due to severe carotid artery stenosis, exacerbated by anemia from cold-induced hemolysis in CAD. Cold exposure precipitated hemolysis, leading to acute anemia and increased blood viscosity, further reducing cerebral blood flow and oxygen delivery. Spontaneous resolution of symptoms upon rewarming supported the role of CAD in her neurological deficits.

This case underscores the potential for CAD to induce neurological deficits in patients with significant carotid artery stenosis. Clinicians should consider CAD as a contributory factor in patients presenting with transient neurological deficits precipitated by cold exposure, especially when significant arterial stenosis is present.

## Introduction

Autoimmune hemolytic anemias (AIHAs) are rare, heterogeneous disorders characterized by the premature destruction of red blood cells (RBCs) due to autoantibodies targeting erythrocyte surface antigens [1]. These autoantibodies are classified based on their thermal reactivity into warm antibodies, which are active at 37°C, and cold antibodies, which are active at lower temperatures. Cold agglutinin disease (CAD), the least common subtype of cold AIHA, is characterized by the presence of high concentrations of circulating antibodies (cold agglutinins), usually immunoglobulin M (IgM), that bind to RBCs specifically at low body temperatures, typically 28-31°C [2,3]. This binding activates the classical complement pathway, leading to complement-mediated intravascular hemolysis via the membrane attack complex [4].

Epidemiological studies from Nordic countries estimate the incidence of CAD at approximately 1 to 1.8 cases per million persons per year, with a prevalence of 13 to 16 cases per million [5,6]. Clinical manifestations of CAD include hemolytic anemia and symptoms related to impaired peripheral circulation, such as acrocyanosis and Raynaud's phenomenon. While CAD-associated hemolysis is known to precipitate venous thromboembolism due to hypercoagulability from free hemoglobin and other prothrombotic factors, its association with arterial thromboembolic events, particularly ischemic stroke, remains controversial and poorly defined [6]. To the best of our knowledge, there are no previous reports directly linking CAD with cerebral infarction.

We present the case of an 84-year-old woman with CAD who experienced recurrent transient ischemic attacks (TIAs) precipitated by cold exposure in the context of significant carotid artery stenosis.

This case was previously presented as a poster at the 20th European Congress of Internal Medicine, held in Malaga, Spain, from June 9 to 11, 2022.

## Case presentation

An 84-year-old woman presented to the emergency department on a cold February morning with the acute onset of left upper extremity weakness. She reported an inability to lift her left arm against gravity and difficulty performing fine motor tasks with her left hand. These symptoms developed shortly after she left her home and was exposed to sub-zero temperatures in her rural village. She also reported generalized fatigue and asthenia.

She recalled a similar episode during the previous winter, characterized by transient left-sided hemiparesis lasting several hours with complete spontaneous resolution. No medical evaluation or intervention was sought at that time. Her medical history was notable for recurrent anemia occurring during the autumn and winter months over the past several years. She had been receiving oral iron supplementation prescribed by her primary care physician, with minimal improvement. On physical examination, the patient was hemodynamically stable, eupneic in ambient air, and afebrile. A neurological assessment revealed decreased muscle strength in the left upper extremity, graded as 4+ out of 5 on the Medical Research Council scale. Fine motor coordination was impaired in the left hand. Sensory examination was normal, and deep tendon reflexes were symmetrical. The cranial nerve examination was intact.

**Figure 1:**
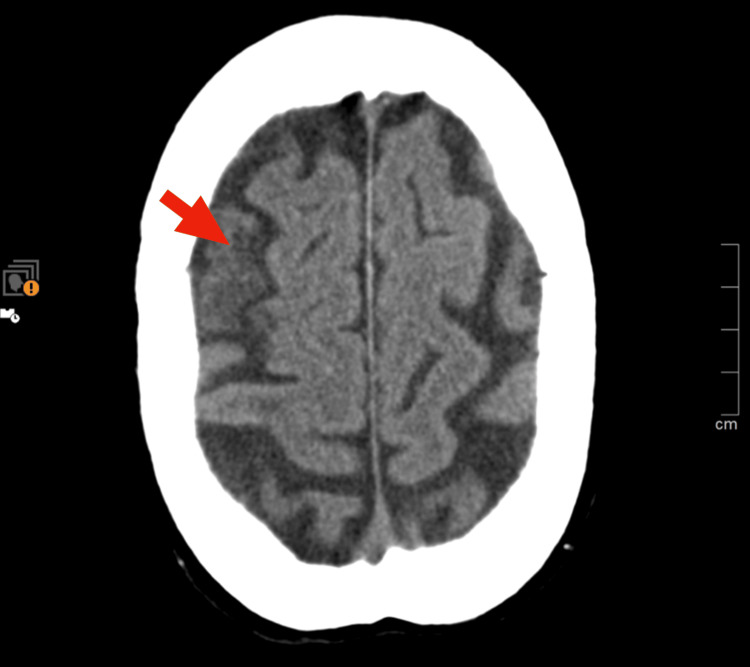
Cranial (CT) scan shows chronic ischemic changes in the right frontocortical and subcortical regions

Scleral icterus was noted, although the patient denied any prior awareness of jaundice. She reported no dark-colored urine or pale stools. There was no lymphadenopathy or hepatosplenomegaly. Skin examination showed no signs of acrocyanosis, Raynaud's phenomenon, or digital ulcers. An urgent non-contrast cranial computed tomography (CT) scan showed no evidence of acute intracranial hemorrhage or infarction. CT angiography of the cerebral vessels revealed no vascular occlusions but demonstrated chronic ischemic changes in the right frontocortical and subcortical regions, consistent with a prior vascular insult. These findings are not responsible for the patient's current symptomatology (Figure 1).

Laboratory investigations revealed normocytic anemia with decreased hemoglobin and hematocrit levels. The mean corpuscular volume (MCV) was within normal limits. Reticulocyte count was elevated, indicating a compensatory bone marrow response. Peripheral blood smear demonstrated erythrocyte agglutination, polychromasia, and anisocytosis. Biochemical tests showed elevated lactate dehydrogenase, elevated total bilirubin with indirect hyperbilirubinemia, and haptoglobin levels reduced below detectable limits. Liver function tests were otherwise normal. The coagulation profile was within normal limits (Table 1). 

**Table 1:** Summary of laboratory results

Test	Result	Normal range
Hemoglobin	9.8 g/dL	Female: 12.0-15.5 g/dL
Hematocrit	30.1%	Female: 36-46%
Mean corpuscular volume	97.8 fL	80-100 fL
Reticulocytes	5%	0.5-2.5%
Haptoglobin	<10 mg/dL	30-200 mg/dL
Leukocytes	5,700 /mm³	4,000-11,000 /mm³
Neutrophils	86.4%	40-70%
Platelets	197,000 /mm³	150,000-450,000 /mm³
International normalized ratio	0.9	0.8-1.2
Urea	58 mg/dL	15-40 mg/dL
Creatinine	1.1 mg/dL	0.6-1.3 mg/dL
Sodium	135 mEq/L	135-145 mEq/L
Potassium	4.8 mEq/L	3.5-5.0 mEq/L
Alkaline phosphatase	106 U/L	44-147 U/L
Gamma-glutamyl transferase	26.4 U/L	9-48 U/L
Alanine aminotransferase	41 U/L	7-56 U/L
Aspartate aminotransferase	35 U/L	10-40 U/L
Lactate dehydrogenase	629 U/L	140-280 U/L
Total bilirubin	6.9 mg/dL	0.2-1.2 mg/dL
Indirect bilirubin	6.23 mg/dL	Calculated (TBil-DBil)
Direct bilirubin	0.67 mg/dL	0.0-0.3 mg/dL

An abdominal ultrasound was performed to evaluate for hepatobiliary causes of jaundice and hemolysis. The liver was of normal size and echotexture, without focal lesions or biliary ductal dilatation. The gallbladder was minimally distended and contained small infundibular gallstones without signs of cholecystitis or biliary obstruction. The spleen was of normal size. The absence of hepatic and biliary tract abnormalities, combined with a spleen size within normal limits, supports the consideration of an intravascular hemolytic etiology.

A direct antiglobulin test (DAT) was positive for complement component C3d and IgM antibodies, confirming the diagnosis of cold agglutinin disease. Cold agglutinin titers were significantly elevated, with a titer of ≥1:64 at 4°C (normal <1:16). Given the partial improvement of her motor deficits and the absence of an occlusive intracranial thrombus, the patient was admitted to the stroke unit for close monitoring and further diagnostic evaluation. During hospitalization, her neurological symptoms resolved completely without specific intervention.

**Figure 2:**
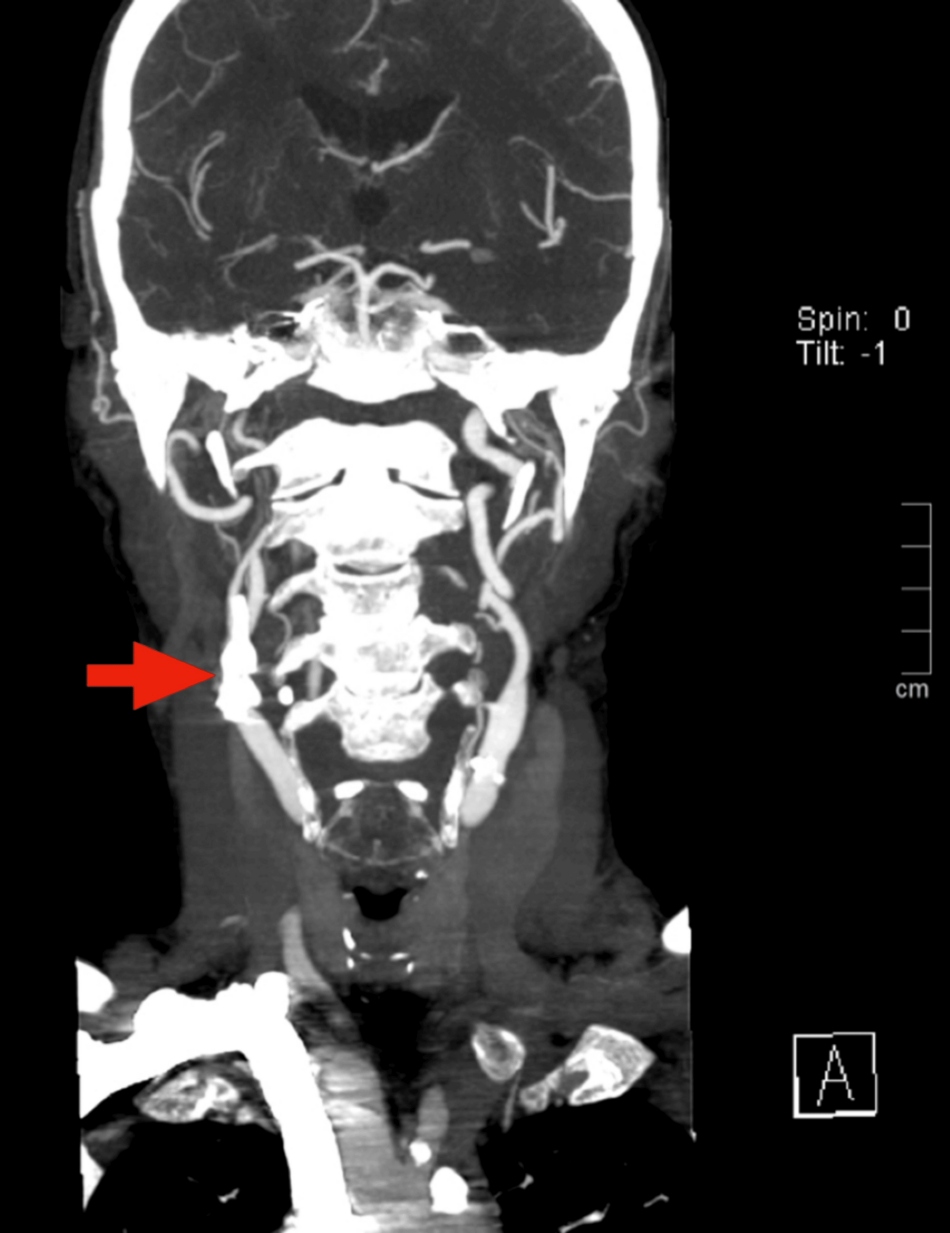
Coronal cervicoencephalic CT angiography shows calcified atherosclerotic plaques in the right carotid artery

**Figure 3:**
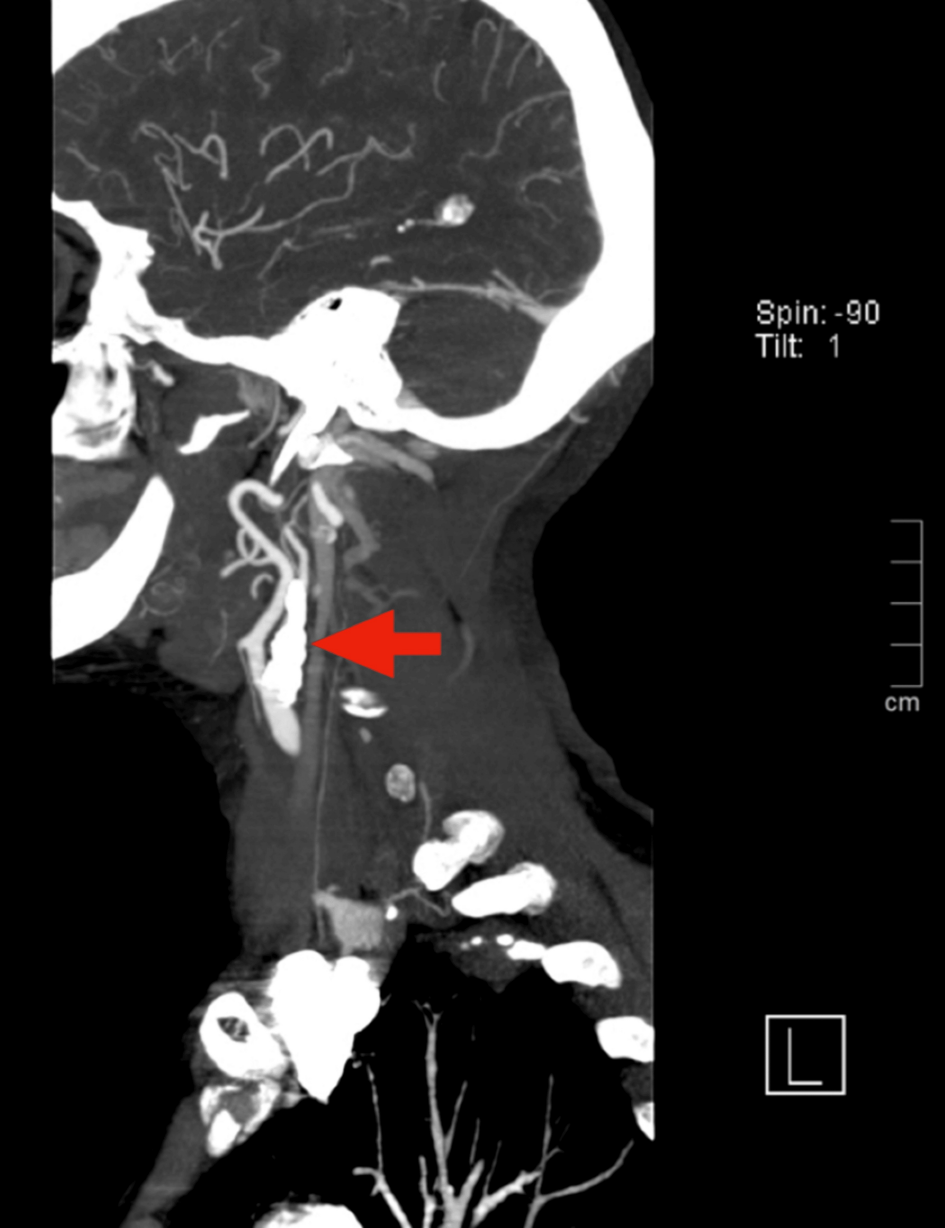
Sagital cervicoencephalic CT angiography shows calcified atherosclerotic plaques in the right carotid artery

**Figure 4:**
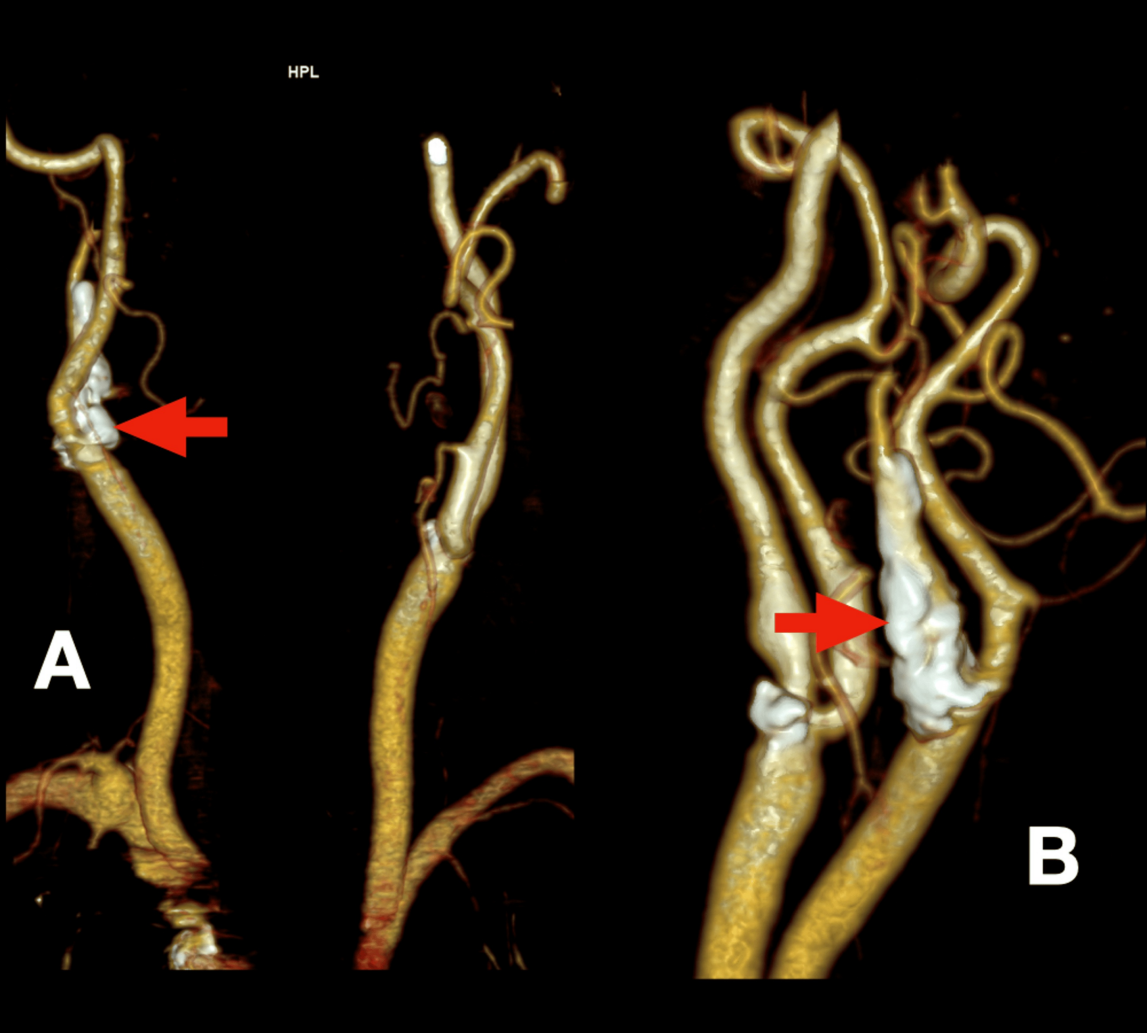
Reconstruction of cervicoencephalic CT angiography Red arrows in panels A and B show, from different perspectives, calcified atherosclerotic plaques involving the bifurcation of the right common carotid artery and the proximal right internal carotid artery

Further evaluation for the transient ischemic attack included carotid duplex ultrasonography, which revealed significant bilateral atherosclerotic disease with moderate calcifications. The right internal carotid artery exhibited greater than 80% stenosis. Cervicoencephalic CT angiography confirmed extensive calcified atherosclerotic plaques involving the bifurcation of the right common carotid artery and the proximal right internal carotid artery, resulting in near-occlusive stenosis of the carotid bulb with diminished distal perfusion (Figures 2-4).

A 24-hour Holter monitor revealed sinus rhythm with a heart rate variability ranging with significant ventricular and supraventricular ectopic activity, including episodes of ventricular bigeminy and supraventricular trigeminy, although the patient remained asymptomatic throughout the monitoring period. Transthoracic echocardiography demonstrated bilateral atrial enlargement, moderate mitral valve regurgitation, normal left ventricular function, and no intracardiac thrombi or septal defects.

Vascular surgery consultation deemed the right carotid artery occlusion to be functionally non-revascularizable due to extensive calcification, near-occlusion, and poor distal vessel quality. Medical management with antiplatelet agents and statins was initiated to reduce the risk of further ischemic events. Hematology consultants recommended conservative management focused on strict avoidance of cold exposure and ensuring adequate thermal insulation year-round. Given the resolution of hemolysis with conservative measures and the patient's advanced age, immunosuppressive therapy or blood transfusions were not pursued.

## Discussion

This case illustrates a rare association between cold agglutinin disease and ischemic cerebrovascular events in the context of significant carotid artery stenosis. CAD is characterized by hemolysis triggered by cold exposure, leading to anemia and peripheral circulatory disturbances. The binding of cold-reactive IgM antibodies to erythrocyte surface antigens activates the classical complement pathway, resulting in complement-mediated intravascular hemolysis via the membrane attack complex [1,2,4]. CAD remains an infrequent subtype of autoimmune hemolytic anemia (AIHA), with a pronounced predilection for female patients [7]. Epidemiological studies consistently demonstrate that the median age at diagnosis predominantly resides within the sixth to seventh decade of life [5]. This demographic trend is exemplified by the current case of an 84-year-old woman, whose advanced age at presentation aligns with established prevalence data.

Clinical manifestations include fatigue, pallor, jaundice, acrocyanosis, Raynaud's phenomenon, and, in severe cases, cutaneous ulceration and necrosis. While CAD-associated hemolysis is known to increase the risk of venous thromboembolism due to hypercoagulability from free hemoglobin and other prothrombotic factors, its association with arterial thromboembolic events remains controversial [6].

In this patient, the severe stenosis of the right internal carotid artery resulted in compromised cerebral perfusion to the right hemisphere. The cold-induced hemolysis led to acute anemia and increased blood viscosity, further reducing cerebral blood flow and oxygen delivery. The spontaneous resolution of her symptoms upon rewarming supports the role of cold exposure and CAD in precipitating her neurological deficits.

Previous imaging findings of chronic ischemic changes in the right frontocortical and subcortical regions suggest prior episodes of cerebrovascular insufficiency, likely exacerbated by recurrent hemolytic events during cold exposure. Management of CAD primarily involves avoidance of cold exposure to prevent hemolysis [8]. Pharmacologic interventions, such as rituximab, have shown efficacy in reducing cold agglutinin titers and ameliorating hemolysis [8]. However, corticosteroids are generally less effective in CAD compared to warm AIHA [7]. In severe cases, plasma exchange or immunosuppressive therapies may be considered. In this case, immunosuppressive therapy was not initiated due to the patient's advanced age, comorbidities, and resolution of hemolysis with conservative measures.

The management of her cerebrovascular disease focused on medical therapy for secondary prevention, as surgical revascularization was not feasible. Antiplatelet agents and statins were prescribed to reduce the risk of further ischemic events. Patient education emphasized the importance of strict cold avoidance, including the use of warm clothing, heated environments, and limiting exposure to cold temperatures.

## Conclusions

This case highlights the potential for cold agglutinin disease to induce neurologic deficits in patients with significant carotid artery stenosis. Clinicians should be cognizant of CAD as a potential contributory factor in patients presenting with transient neurological deficits precipitated by cold exposure, particularly when significant arterial stenosis is present. The cold-induced hemolysis characteristic of CAD leads to acute anemia and increased blood viscosity, thereby further compromising cerebral blood flow and oxygen delivery. Comprehensive evaluation, including hematological and vascular assessments, is essential for accurate diagnosis and tailored treatment, which may range from cold avoidance strategies and corticosteroid therapy to rituximab administration or vascular interventions. Further research is needed to elucidate the pathophysiological mechanisms connecting CAD with arterial ischemia and to develop tailored treatment strategies for patients with concomitant cerebrovascular disease.
